# The complete mitochondrial genome of the bagworm from a tea plantation in China, *Eumeta variegata* (Lepidoptera: Psychidae)

**DOI:** 10.1080/23802359.2021.1886009

**Published:** 2021-03-15

**Authors:** Shi-Chun Chen, Feng-Hua Zhao, Hong-Yan Jiang, Xiang Hu, Xiao-Qing Wang

**Affiliations:** a Tea Research Institute of Chongqing Academy of Agricultural Science, Chongqing, PR China; b Xinyang Academy of Agricultural Sciences, Xinyang, PR China

**Keywords:** Mitochondrial genome, *Eumeta variegata*, bagworm moth, phylogeny

## Abstract

The mitochondrial (mt) genome of *Eumeta variegata* Snellen (Psychidae) has been sequenced and annotated. The mt genome has a total length of 15,793 bp, consisting of 13 protein-coding genes, 22 tRNA, two rRNA genes, and an AT-rich control region (GenBank accession no. MN242985). The nucleotide composition was extremely AT-rich, and the AT content is 81.57%. The gene order is consistent with other sequenced mt genome of moths and butterflies from Ditrysia. The sequence similarity of *E. variegate* mt genomes between the specimen of China and South Korea is 98.38%, whereas the similarity between the specimen of China and Japan is 90.61%. The sequence of PCGs and rRNAs among different specimens are similar, and many differences are detected at the region of A + T-rich region and the tRNA block ‘ARNS_1_EF’.

The bagworm *Eumeta variegata* Snellen (synonyms for *Eumeta japonica*, *Clania variegate*), belongs to the family Psychidae in the superfamily Tineoidea (Nishida 1983; Sugimoto 2009). The larvae of *Eumeta variegate* consume tea, citrus, coffee, loquat, pear, peach, and other plants, influencing the quality and quantity of farm produce, causing considerable economic losses. Larvae of *E. variegata*, collected from a tea plantation in Enshi, Hubei, China (N30°19′, E109°28′), were identified to species by morphology (Sugimoto 2009; Zhang 2010). Voucher specimens (#CQNKY-LE-02-02-01) were deposited at the Insect Collection, Tea Research Institute of Chongqing Academy of Agricultural Science, Chongqing, China. Total genomic DNA was extracted using a Dneasy Blood and Tissue Kit (Qiagen, Hilden, Germany) and stored at −20 °C. PCR amplification method was used to amplify the mt genome sequence of *E. variegate*. Partial sequences of *cox1*, *cox2*, *nad4*, *nad5*, *cob*, *rrnS*, and *rrnL* genes were amplified initially by PCR with conserved insect primers (Liu and Beckenbach 1992; Simon et al. 1994; Zhang et al. 2014), and long-PCR primers were designed from them.

The complete mitochondrial (mt) genome of *E. variegata* from China is 15,793 bp (GenBank accession no. MN242985). The mt genome encodes all 37 genes usually found in animal mt genomes, including 13 protein-coding genes (PCGs), two ribosomal RNAs, and 22 transfer RNAs. The gene arrangement is identical to that of other bagworm and Distrysian moths generally (Li et al. 2017). The nucleotide composition was extremely AT-rich, and the AT content is 81.57%.

The complete mt genome of this species has been reported from Japan (Arakawa et al. 2018) and South Korea (Jeong et al. 2018) where the genome size was 16,601 bp and 15,660 bp, respectively. The total sequence similarity of mt genomes between the specimen described in this study and that from South Korea (Jeong et al. 2018) is 98.38%. The sequence similarities of PCGs are high, ranging from 99.43% (*atp8*) to 100% (*cox3* and *nad4L*), rRNA sequence similarities are lower than that of PCGs, at values of 99.25% (*rrnL*) and 96.68% (*rrnS*). Between this specimen from China and Japan (Arakawa et al. 2018), the overall sequence similarity is 90.61%, PCG sequence similarities are ranging from 98.96% (*cox1*) to 99.43% (*atp8*), and rRNA sequence similarities are 96.52% (*rrnL*) and 95.49% (*rrnS*). Among three specimens, two mt genomes of *E. variegate* from China and South Korea have a higher sequence identity, a similar size, and the same gene content. The mt genome of *E. variegate* from Japan has a larger A + T-rich region (1118 bp), and the other two have shorter A + T-rich regions, 94 bp (South Korea) and 86 bp (China). The two short A + T-rich region sequences were well-aligned at the beginning and end regions with the larger one. The mt genome of *E. variegate* from Japan contained only 20 tRNAs without *trnE* and *trnF*, and a 378 bp long non-coding sequence located between *trnS_1_* and *nad5* instead of *trnE* and *trnF*. Furthermore, the great differences between the mt genomes of *E. variegate* from China and South Korea are also detected in the region of gene block ‘*trnA*-*trnR*-*trnN*-*trnS_1_*-*trnE*-*trnF*’ (tRNA block ‘ARNS_1_EF’), that locates between genes *nad3* and *nad5* (Figure 1(B)). The intergenic spacers were varied in length due to the simple repeats.

**Figure 1.**
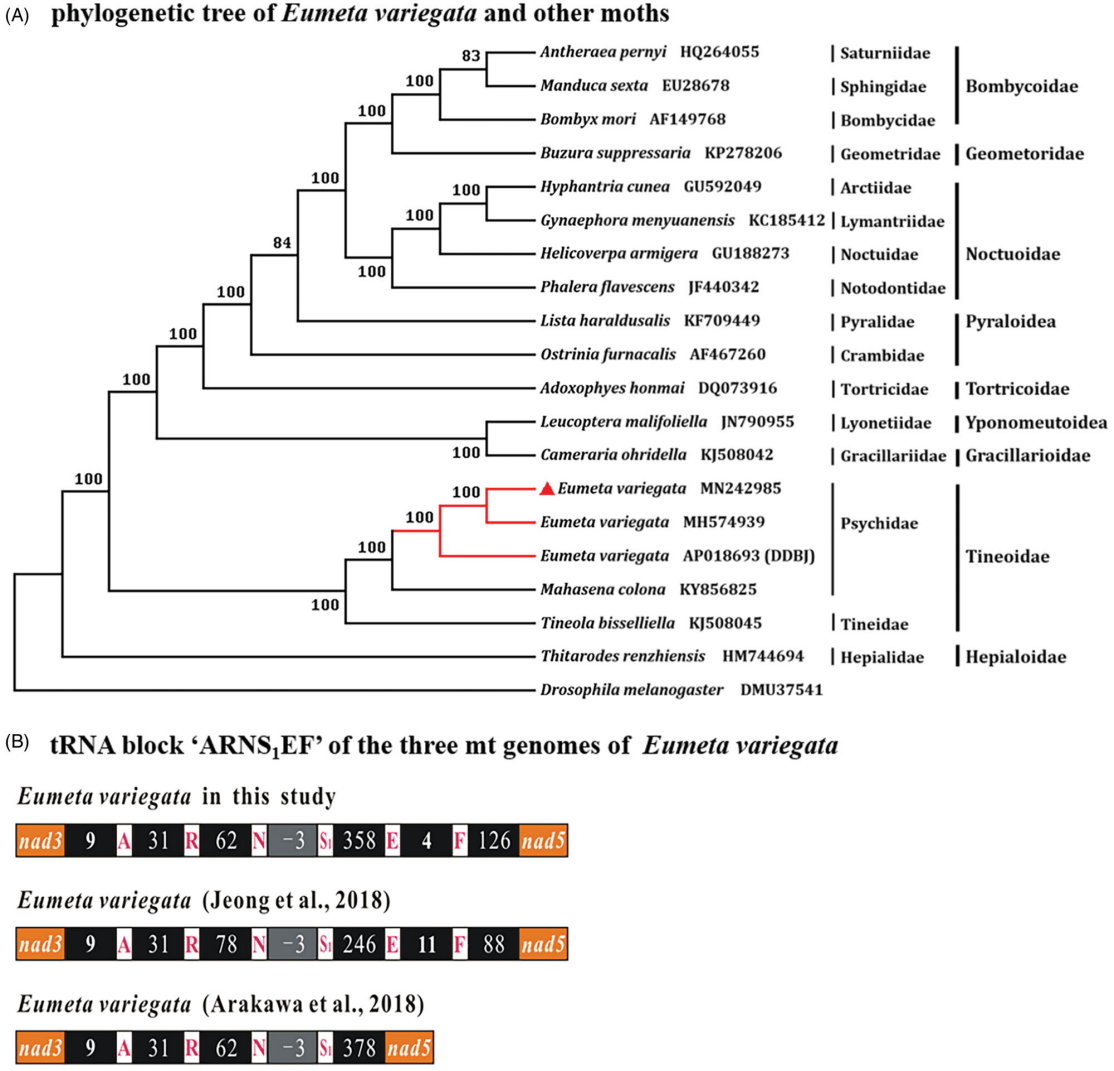
The maximum-likelihood (ML) phylogenetic tree and tRNA block ‘ARNS_1_EF’ of the three mt genomes of *Eumeta variegata*. nad3 and nad5 are protein-coding genes, single capital letters are standard abbreviations of tRNA gene, intergenic spacers and gene overlaps shown in numbers. Codon of trnS1 is AGN.

We analyzed 13 PCGs with maximum-likelihood (ML) method utilizing PhyML3.0 (http://www.atgc-montpellier.fr/phyml/) to explorer the phylogenetic relationship of *E. variegate* with other moths. The mt genome sequence of *Drosophila melanogaster* (GenBank accession no. DMU37541) was used as an outgroup. In the phylogenetic tree, two specimens of *E. variegate* from China and South Korea were clustered together, and formed a sister clade to the specimen from Japan. Three Tineidae species (*E. variegate*, *M. colona*, and *Tineola bisselliella*) were monophyletic with 100% bootstrap value (Figure 1(A)). The suborder Ditrysia was monophyletic with Tineoidea sister to the remaining Ditrysia (Timmermans et al. 2014).

## Data Availability

The data that support the findings of this study are openly available in GenBank of NCBI at https://www.ncbi.nlm.nih.gov, reference number MN242985.
